# Metabolic, physiological and anatomical responses of soybean plants under water deficit and high temperature condition

**DOI:** 10.1038/s41598-022-21035-4

**Published:** 2022-10-01

**Authors:** Roberto Gomes Vital, Caroline Müller, Francisco Bruno Silva Freire, Fábia Barbosa Silva, Priscila Ferreira Batista, David Fuentes, Arthur Almeida Rodrigues, Luciana Minervina Freitas Moura, Danilo Menezes Daloso, Adinan Alves Silva, Andrew Merchant, Alan Carlos Costa

**Affiliations:** 1 Laboratório de Ecofisiologia e Produtividade Vegetal, Instituto Federal Goiano – Campus Rio Verde, Caixa Postal 66, Rio Verde, GO 75901-970 Brazil; 2 LabPlant, Departamento de Bioquímica e Biologia Molecular, Universidade Federal do Ceará, Fortaleza, CE 60451-970 Brazil; 3 Centre for Carbon Water and Food, The University of Sydney, 380 Werombi Road, Camden, NSW 2570 Australia; 4 Laboratório de Anatomia Vegetal, Instituto Federal Goiano – Campus Rio Verde, Caixa Postal 66, Rio Verde, GO 75901-970 Brazil; 5 Centro de Excelência em Agricultura Exponencial (CEAGRE), Rio Verde, Rio Verde, GO 75901-970 Brazil

**Keywords:** Plant physiology, Abiotic, Drought, Heat

## Abstract

Water deficit (WD) combined with high temperature (HT) is the major factor limiting agriculture worldwide, and it is predicted to become worse according to the current climate change scenario. It is thus important to understand how current cultivated crops respond to these stress conditions. Here we investigated how four soybean cultivars respond to WD and HT isolated or in combination at metabolic, physiological, and anatomical levels. The WD + HT increased the level of stress in soybean plants when compared to plants under well-watered (WW), WD, or HT conditions. WD + HT exacerbates the increases in ascorbate peroxidase activity, which was associated with the greater photosynthetic rate in two cultivars under WD + HT. The metabolic responses to WD + HT diverge substantially from plants under WW, WD, or HT conditions. *Myo*-inositol and maltose were identified as WD + HT biomarkers and were connected to subnetworks composed of catalase, amino acids, and both root and leaf osmotic potentials. Correlation-based network analyses highlight that the network heterogeneity increased and a higher integration among metabolic, physiological, and morphological nodes is observed under stress conditions. Beyond unveiling biochemical and metabolic WD + HT biomarkers, our results collectively highlight that the mechanisms behind the acclimation to WD + HT cannot be understood by investigating WD or HT stress separately.

## Introduction

Soybean (*Glycine max* (L.) Merr.) is an economically important crop worldwide. Breeding programs over the last century have resulted in highly productive soybean cultivars that are adapted to distinct environmental conditions. However, increasing climate instability, which is predicted to directly alter both rainfall and environmental temperature, has raised concerns regarding the maintenance of soybean crop yield^1–3^. Periodic temperature increases at critical stages of soybean development associated with lower rainfall have been reported in various continents. In Brazil, one of the major soybean producers, the reduced yield of soybean cultivated in the Central-West region in 2018/2019 was associated with an increase of up to 3 °C above the average temperature normally found in this area^4,5^. This highlights how climate change may affect soybean production in tropical areas, which has clear impacts worldwide. Thus, understanding the mechanisms by which plants acclimate to water deficit (WD) combined with high temperature (HT) is crucial for identifying stress tolerant cultivars and to pave the way for obtaining soybean cultivars with higher productivity under combined WD and HT stress conditions.

Plant stress responses involve the modulation of a complex network that leads to molecular, physiological, and/or morphological alterations in the plant according to the intensity of the stress level^6,7^. In general, plant acclimation to WD initially includes the activation of mechanisms such as abscisic acid-mediated stomatal closure and osmotic adjustment through the accumulation of several osmoprotective compounds, such as sugars, sugar-alcohols, and amino acids^8–12^. The combination of these and several other hormonal, hydraulic, signaling, and antioxidant mechanisms triggered by WD are important to minimize deleterious effects caused by the stress and to maintain cell turgor and plant growth^13–15^. Under HT conditions, sucrose accumulation has also been described as an important component of osmotic adjustment^16^. Furthermore, it has been shown that amphiphilic molecules such as proline and sugar alcohols act in the stabilization of cellular structures protecting cell membranes affected by heat stress^17^. It seems likely therefore that plant responses to heat stress have certain similarities with drought. However, drought is commonly associated with HT under field conditions and evidence highlight that the metabolic responses to the combination of both stresses are substantially different from drought or heat stress separately^18^. It is thus important to investigate both stresses combined to unveil which mechanisms confer higher plasticity or higher tolerance to the combination of WD and HT stresses.

Soil water availability is generally reduced as temperature increases. Under these conditions, stomatal closure is an important response to avoid excessive water loss, but as consequence, this reduces CO_2_ fixation mediated by ribulose-1,5-bisphosphate carboxylase/oxygenase activity^19^. Additionally, thermal stress reduces CO_2_ solubility and increase the permeability of the thylakoid membranes, favoring O_2_ fixation and thus photorespiration and destabilizing the photosynthetic electron transport chain^20^. This leads to an energy imbalance promoted by excessive light absorption, which increases the production of oxidizing compounds such as reactive oxygen species (ROS), causing oxidative damage to lipids and membrane proteins^21^. As a defense mechanism, plants acclimate to combined WD and HT activating enzymatic mechanisms of the antioxidant defense system to remove excess ROS. This includes an increase in the activity of superoxide dismutase (SOD), catalase (CAT), ascorbate peroxidase (APX), and peroxidase (POD)^22^ as well as the activation of non-enzymatic ROS scavenging mechanisms such as the synthesis of ascorbate^23^ and flavonoids/anthocyanins^24^. These mechanisms have been highlighted as important to the tolerance of HT and WD by plants^25^. For instance, increased antioxidant enzyme activity was observed in the WD tolerant canola variety^26^ and in heat stress-tolerant chicken grass plants^27^. However, it is still unclear whether the antioxidant system is an active response or a passive consequence of soybean acclimation to WD + HT.

Several examples in the literature highlight that identifying drought stress tolerant genotypes is commonly achieved at the expense of photosynthetic rate, growth, and/or yield under non-adverse and/or stress conditions^28–30^. However, given current and projected demand for food production, genotypes that maintain high photosynthetic performance under the combination of WD and HT without severe penalties in yield under both non-adverse and stressed conditions are desirable^31^. The major challenge for plant scientists is thus to fully comprehend the trade-off among yield and (a)biotic stress tolerance in order to successfully manipulate plant metabolism toward simultaneous plant growth and stress tolerance improvements^32,33^. This challenge is higher when the combination of WD and HT is considered, given that the effects of these stresses combined can exacerbate damage to metabolism, reducing crop growth and yield^34,35^. Furthermore, studies integrating physiological, morphoanatomical, and metabolic responses of plants exposed to combined stress are scarce, which hampers our understanding concerning the modulation of the entire plant network under WD + HT. In order to address this gap, we used an integrative approach to investigate the physiological, morphoanatomical, and metabolic responses of four soybean cultivars subjected to WD and HT in isolated or combined conditions. The results are discussed in the context of the importance in considering investigating WD and HT combined to unveil the mechanisms by which plants acclimate under these stress conditions.

## Results

### The combination of water deficit and high temperature exacerbates stress-induced physiological changes

**Figure 1:**
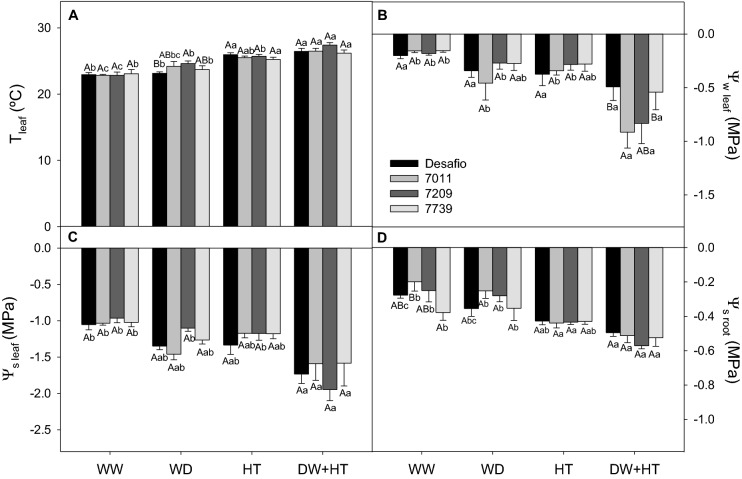
Leaf temperature and hydric status of soybean plants maintained for 8 days under well-watered (WW; 100% holding water capacity HWC, 25 °C), water deficit (WD; 40% HWC, 25 °C), high temperature (HT; 100% HWC, 40 °C), and water deficit plus high temperature (WD + HT; 40% HWC; 40 °C) conditions. (**A**) Leaf temperature (°C). (**B**) Leaf water potential (Ѱ_w(am)_). (**C**) Leaf osmotic potential (Ѱ_s(leaf)_). (**D**) Root osmotic potential (Ψ_s(root)_). Bars represent mean ± SE (*n* = 5). Means followed by the same uppercase letters compare cultivars within the same water and temperature treatment. Means followed by the same lowercase letters compare water and temperature treatments within the same cultivar, as determined by the Tukey test at 5% of probability.

**Figure 2:**
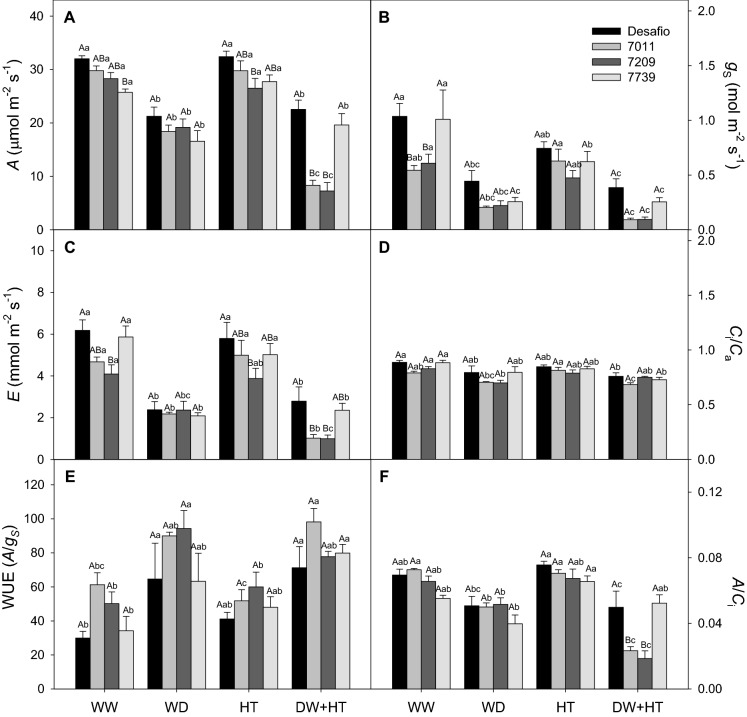
Gas exchange parameters of soybean plants maintained for 8 days under well-watered (WW; 100% holding water capacity HWC, 25 °C), water deficit (WD; 40% HWC, 25 °C), high temperature (HT; 100% HWC, 40 °C), and water deficit plus high temperature (WD + HT; 40% HWC; 40 °C) conditions. (**A**) Photosynthetic rate (*A*, µmol m^−2^ s^−1^). (**B**) Stomatal conductance (*g*_s_, mol m^−2^ s^−1^). (**C**) Transpiration rate ((**E**), mmol m^−2^ s^−1^). (**D**) Intrinsic water-use efficiency (WUE = *A*/*g*_s_). (**E**) Ratio of internal and external CO_2_ concentration (*C*_*i*_*/C*_*a*_). (**F**) Instantaneous carboxylation efficiency (*A*/*C*_i_,). Bars represent mean ± SE (*n* = 5). Means followed by the same uppercase letters compare cultivars within the same water and temperature treatment. Means followed by the same lowercase letters compare water and temperature treatments within the same cultivar, as determined by the Tukey test at 5% of probability.

Increases in leaf temperature were recorded under all stress treatments, with increments up to 3.71 °C in WD + HT-treated plants compared to WW plants (Fig. 1A). Lower average values of Ψ_w(am)_ (− 0.7 MPa) Ѱ_s(leaf)_ (− 1.7 MPa), and Ѱ_s(root)_ (− 0.5 MPa) were observed in plants under WD + HT compared to the WW condition, with no major difference among the cultivars under this condition (Fig. 1B–D). WD and WD + HT reduced *A*, *g*_s,_ and *E* in all soybean cultivars when compared to their respective controls, but this was not reflected in *C*_i_/*C*_a_ ratio (Fig. 2A–D). The 7011 and 7209 cultivars showed the lowest values of *A* (8.3 µmol m^−2^ s^−1^), *g*_s_ (0.09 mol m^−2^ s^−1^), and *E* (1.02 mmol m^−2^ s^−1^) under WD + HT than the cultivars Desafio and 7739 (Fig. 2A–C). The intrinsic water-use efficiency (iWUE—*A*/*g*_s_) increased in WD and WD + HT treatments, but no major differences among the cultivars were observed (Fig. 2E). The *A*/*C*_i_ ratio decreased (~ 70%) mainly in 7011 and 7209 cultivars under WD + HT compared to WW plants (Fig. 2F). The impact of the stress on gas exchange parameters did not resemble those related to leaf temperature and hydric status. It appears that the reductions in gas exchange parameters observed under WD + HT are most associated with WD rather than the HT effect.

**Figure 3:**
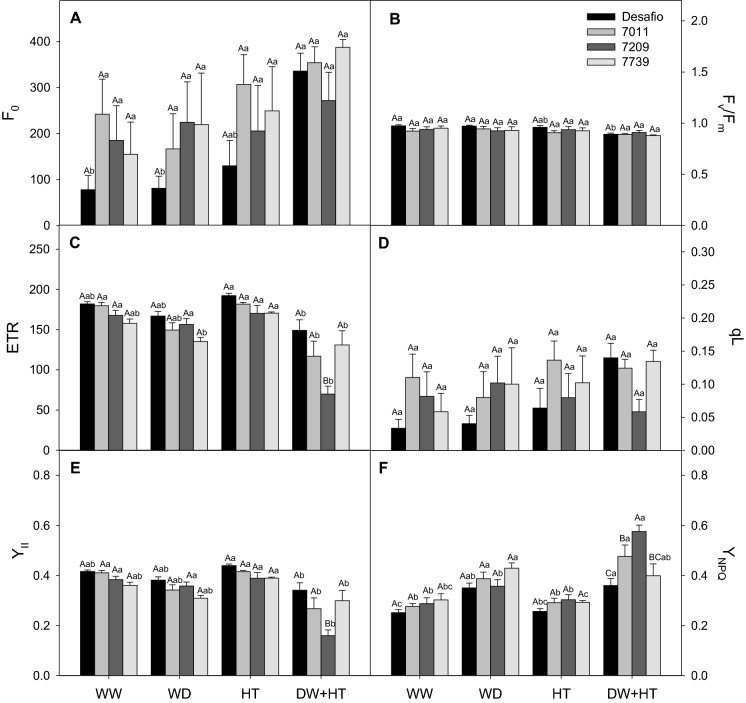
Photochemical parameters of soybean plants maintained for 8 days under well-watered (WW; 100% holding water capacity HWC, 25 °C), water deficit (WD; 40% HWC, 25 °C), high temperature (HT; 100% HWC, 40 °C), and water deficit plus high temperature (WD + HT; 40% HWC; 40 °C) conditions. (**A**) Minimal chlorophyll fluorescence (F_0_). (**B**) Maximum quantum yield of photosystem II (PSII) (Fv/Fm). (**C**) Electron transport rate (ETR). (**D**) Fraction of opened PSII reaction centers (qL). (**E**) Effective quantum yield of PSII (Y_II_). (**F**) Yield of non-photochemical quenching (Y_NPQ_). Bars represent mean ± SE (*n* = 5). Means followed by the same uppercase letters compare cultivars within the same water and temperature treatment. Means followed by the same lowercase letters compare water and temperature treatments within the same cultivar, as determined by the Tukey test at 5% of probability.

The minimum fluorescence (F_0_) presented the highest value (354.6) in cultivar 7011, while the and the maximum quantum yield of PSII (F_v_/F_m_) showed the lowest value (0.88) in 7739 in response to WD + HT, compared to the cultivars Desafio and 7739 (Fig. 3A,B). The electron transport rate (ETR), coefficient quenching (qL), the effective quantum yield of PSII (Y_II_), and the yield of non-photochemical quenching (Y_NPQ_) showed a significant interaction between cultivation conditions and cultivars, where the cultivar 7209 had the lowest values for ETR (69.8), Y_II_ (0.16) and qL (0.06), and the highest value for Y_NPQ_ (0.58) when exposed to WD + HT treatment (Fig. 3). Y_II_ and ETR values were also reduced due to combined stress for cultivars 7011 (Fig. 3C,E). Y_NPQ_ increased for Desafio (0.26), 7011 (0.29), and 7739 (0.29) cultivars in response to HT treatment (Fig. 3F). Under WD + HT, all cultivars increased Y_NPQ_ but were more pronounced for 7209 (0.58) and 7011 (0.48) (Fig. 3F).

### Antioxidant enzyme activity under stress conditions

**Figure 4:**
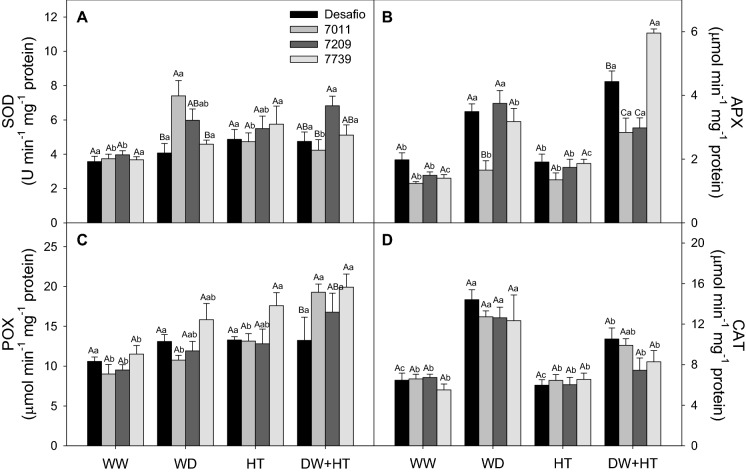
Activity of antioxidant enzymes of soybean plants maintained for 8 days under well-watered (WW; 100% holding water capacity HWC, 25 °C), water deficit (WD; 40% HWC, 25 °C), high temperature (HT; 100% HWC, 40 °C) and water deficit plus high temperature (WD + HT; 40% HWC; 40 °C) conditions. (**A**) Superoxide dismutase (SOD). (**B**) Ascorbate peroxidase (APX). (**C**) Total peroxidase (POX). (**D**) Catalase Bars represent mean ± SE (*n* = 5). Means followed by the same uppercase letters compare cultivars within the same water and temperature treatment. Means followed by the same lowercase letters compare water and temperature treatments within the same cultivar, as determined by the Tukey test at 5% of probability.

SOD activity was higher in cultivar 7011 under WD (5.98 U min^−1^ mg^−1^ protein), cultivar 7739 under HT (5.76 U min^−1^ mg^−1^ protein), and cultivar 7209 exposed to WD + HT (6.83 U min^−1^ mg^−1^ protein) (Fig. 4A). Under WD, the Desafio, 7209, and 7739 cultivars presented higher APX activities, on average (3.47 µmol min^−1^ mg^−1^ protein), while in the WD + HT treatment the Desafio (4.43 µmol min^−1^ mg^−1^ protein) and 7739 (5.96 µmol min^−1^ mg^−1^ protein) cultivars had higher values of APX activity(Fig. 4B). Lower APX activity was observed in the cultivar 7011 (1.66 µmol min^−1^ mg^−1^ protein) under WD condition. POX activity increased in all treatments, especially in WD + HT, on average 17.30 µmol min^−1^ mg^−1^ protein (Fig. 4C). Greater CAT activities were observed in all cultivars exposed to WD, on average, 13.03 µmol min^−1^ mg^−1^ protein, followed by WD + HT treatment (9.04 µmol min^−1^ mg^−1^ protein) (Fig. 4D). In summary, the activity of antioxidant enzymes differs among the stress conditions, in which APX and POX activities increased mainly under WD + HT, and CAT activity increased mainly under WD condition.

### Impact of water deficit combined with high temperature on leaf anatomy and plant growth

HT increased the thickness of palisade parenchyma, on average 104.5 µm, and spongy parenchyma, on average 94.9 µm, in Desafio and 7011 cultivars, which leads to an increase in mesophyll cells (203.5 µm) and leaf thickness (229.2 µm), mainly in 7011 (Supplementary Fig. S1). WD + HT treatment reduced mesophyll (138.2 µm), and palisade (74.4 µm) and spongy (66.1 µm) parenchymas thickness in the cultivar 7011 (Supplementary Fig. S1A–C), and resulted in smaller leaf thickness (159.5 (74.4 µm) compared to the other cultivars (Supplementary Fig. S1D). Higher adaxial (18.8 µm) and abaxial (15.7 µm) epidermis thickness were observed for the cultivar 7011 and lower values for 7209, on average 13.6 µm, both exposed to WD treatment (Supplementary Fig. S1E,F).

Plant height (PH) showed lower values when exposed to WD and WD + HT (Supplementary Fig. S2A), being more pronounced in Desafio and 7739 cultivars. HT treatment promoted an increase in stem diameter (SD, Supplementary Fig. S2B) in 7011 (7.22 mm) and 7739 (6.44 mm). Cultivars 7011 (1111.2 cm^2^) and 7739 (1586.0 cm^2^) presented larger leaf areas (LA) when subjected to HT (Supplementary Fig. S2C), while LA was reduced in cultivar 7011 exposed to WD (713.9 cm^2^). Shoot dry matter (SDM) was increased in cultivars 7011 (10.9 g) and 7739 (8.8 g) in HT condition and reduced in cultivar 7011 (4.15 g) under WD + HT (Supplementary Fig. S2D). Reductions in root dry mass (RDM) were observed in all treatments (Supplementary Fig. S2E), while RDM/SDM was reduced only in HT- and WD + HT-treated plants, with values on average 0.24 g and 0.17 g, respectively (Supplementary Fig. S2F).

### Soybean metabolic responses to water deficit, high temperature, and the combination of both stresses

**Figure 5:**
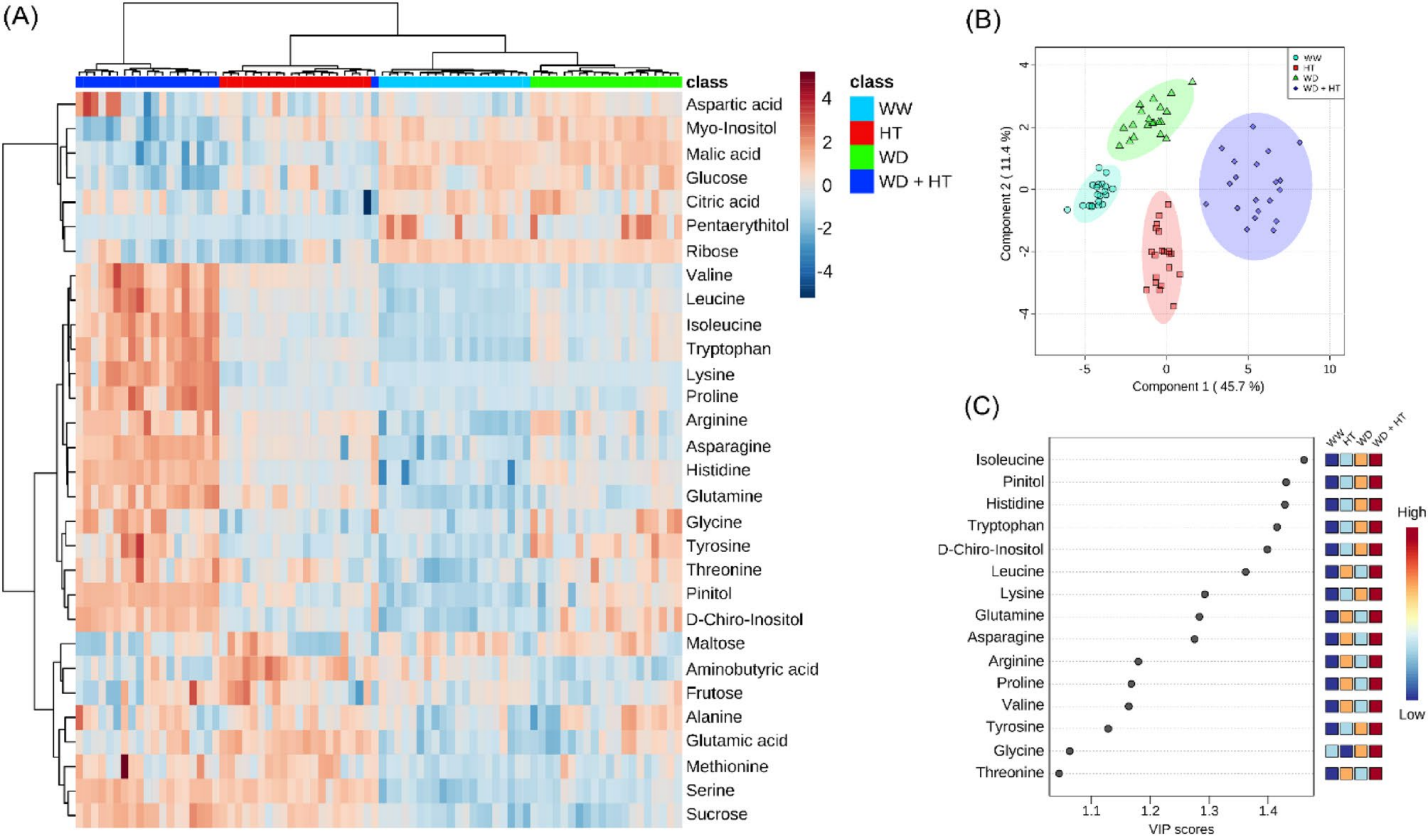
Metabolic characterization of soybean plants maintained for 8 days under well-watered (WW; 100% holding water capacity HWC, 25 °C), water deficit (WD; 40% HWC, 25 °C), high temperature (HT; 100% HWC, 40 °C) and water deficit plus high temperature (WD + HT; 40% HWC; 40 °C) conditions. (**A**) Heat map representation of the content of the metabolites identified in this study. (**B**) Partial least square-discriminant analysis (PLS-DA). (**C**) Variable importance in projection (VIP) scores of the PLS-DA model. Metabolites included in this VIP score list have score higher than 1, which indicates those that mostly contributed to the separation observed at the PLS-DA model. PLS-DA was carried out by combining data from all genotypes. The data were normalized by using Log and Auto-scaling transformations on the MetaboAnalyst platform^98^ (*n* = 5).

Thirty metabolites pertaining to the classes of sugars, amino acids, organic acids, and others were identified in all treatments (Fig. 5A) and used for multivariate and correlation-based network analyses. The partial least square discriminant analysis (PLS-DA) combining the data of each treatment from all cultivars revealed that WD and HT treatments were slightly separated from WW, whilst WD + HT is clearly the most different treatment at the metabolic level. This is evidenced by the separation of WD + HT from the other treatments by the first component, which represents 45.8% of the variability of the model (Fig. 5B). Fifteen metabolites were found with VIP scores higher than 1, meaning that they greatly contribute to the discrimination found in the PLS-DA model. Most of them are amino acids and have higher accumulation in WD + HT compared to WW, WD, and HT treatments (Fig. 5C). Surprisingly, very few significant correlations were observed among the metabolites in each treatment, leading to correlation-based networks that were poorly connected (Supplementary Fig. S4). However, WD and WD + HT have metabolic networks with higher density, as compared to WW (Supplementary Table S1). Additionally, increased network heterogeneity and decreased network centralization were observed in all stress treatments compared to WW (Supplementary Table S1).

**Figure 6:**
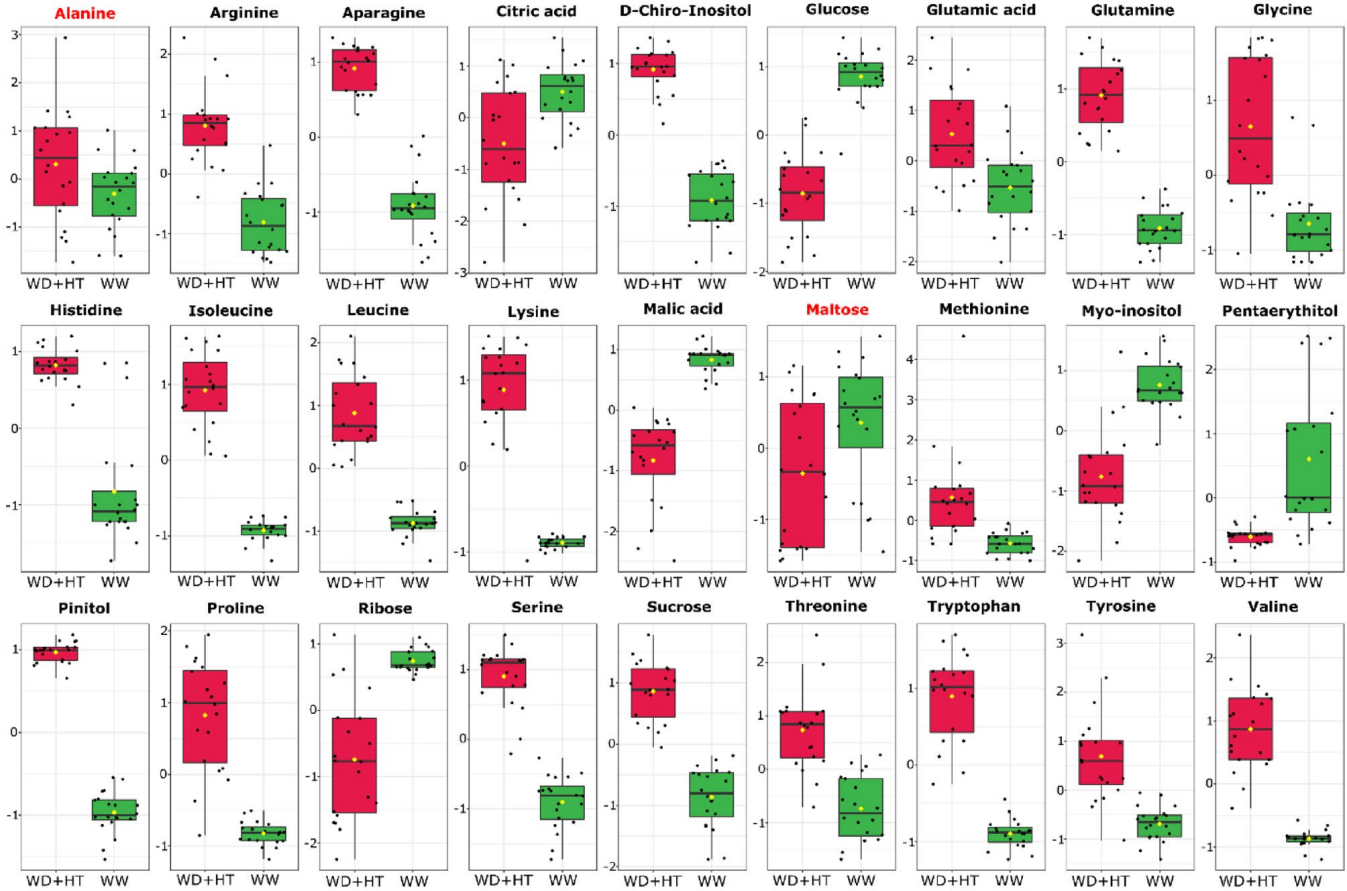
Box plots of metabolites identified as water deficit combined with high temperature (WD + HT) biomarkers. Green and red box plots indicate the relative content of metabolites found in soybean plants under well-watered (WW) and WD + HT conditions, respectively. Biomarkers were identified based on receiver operating characteristic (ROC) curves using Log and Auto-scaling normalized data on the MetaboAnalyst platform^98^. Metabolites in black and red indicate statistical difference at *p* < 0.001 and *p* < 0.05, respectively.

We next investigated which metabolites are biomarkers of each stress condition. A total of 21, 23, and 27 metabolites were identified as biomarkers of WD, HT, and WD + HT stress conditions, respectively (Fig. 6; Supplementary Figs. S5, S6). Among them, only ribose was identified as a biomarker with decreased content in all stress conditions (Supplementary Fig. S7), whilst several amino acids plus pinitol and D-chiro-inositol were identified as biomarkers and with increased content in all stress conditions (Supplementary Fig. S7). Interestingly, *myo*-inositol was found as WD and WD + HT biomarker, but with increased and decreased content in WD and WD + HT, respectively, compared to WW conditions (Supplementary Fig. S7). Similarly, GABA (gamma-aminobutyric acid) was simultaneously found as a biomarker of both HT and WD, but with increased and decreased content under HT and WD, respectively (Supplementary Fig. S7). Sucrose and proline, two important osmoprotective compounds, were found as biomarkers with increased content under both HT and WD + HT, compared to WW conditions (Supplementary Fig. S7).

### Cultivar-specific stress metabolic responses

We next analysed the effect of the stress treatments in each cultivar at metabolic level. The PLS-DA results resemble the general ones described above, in which WD + HT treatment is clearly separated from the other treatments in each cultivar (Fig. 6A–D). Regarding the VIP scores of this analysis, 15, 16, 16, and 14 metabolites have VIP scores higher than 1 in 7011, 7209, 7739, and Desafio cultivars, respectively (Fig. 6E–H). Ten of these metabolites are common among all cultivars, including seven amino acids (His, Leu, Ile, Val, Trp, Asn, and Gln) plus pinitol, sucrose, and glucose (Supplementary Fig. S8).

### Integrating metabolomics with physiological parameters

**Figure 7:**
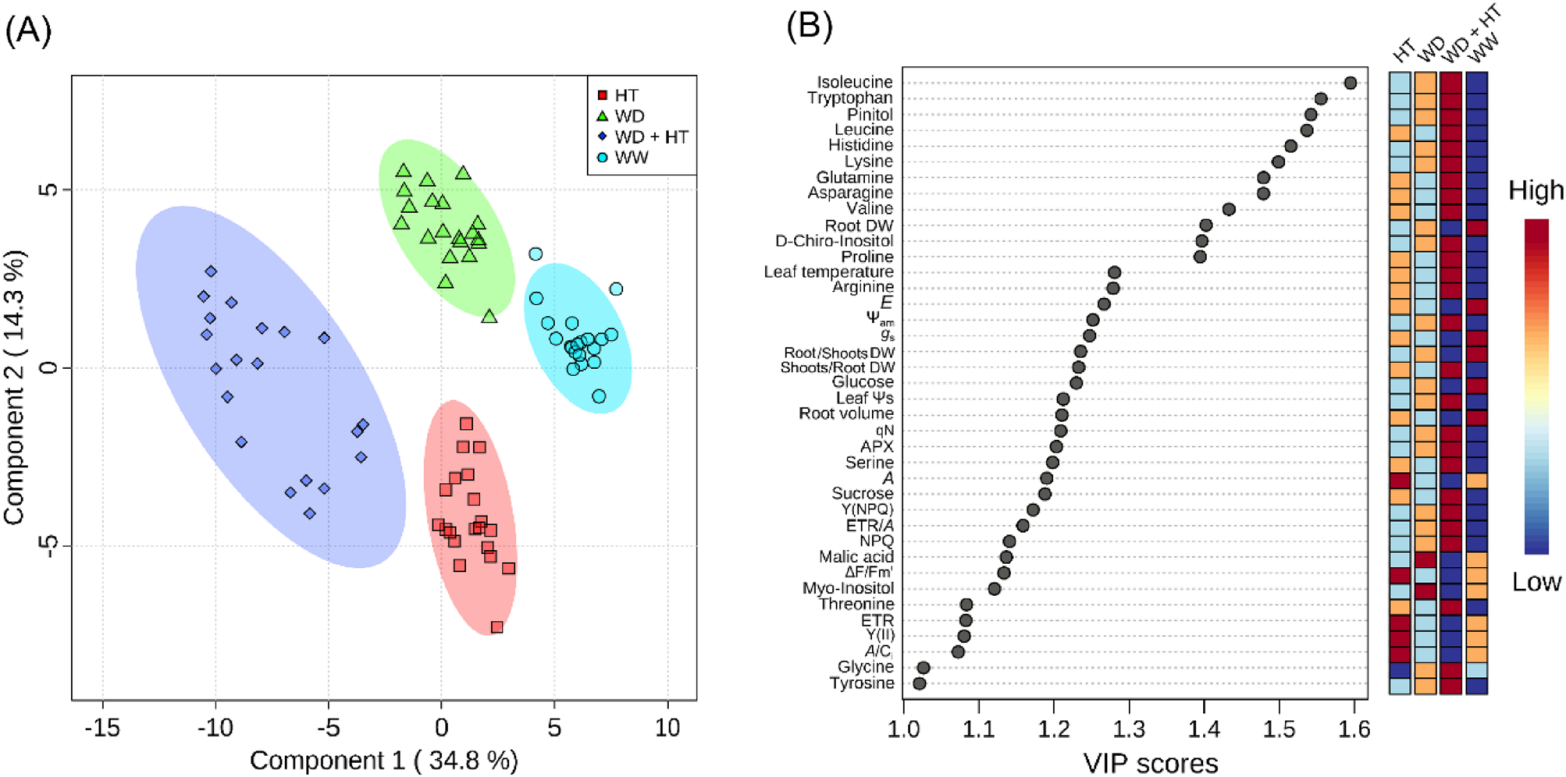
Multivariate analysis combining all data of soybean plants maintained for 8 days under well-watered (WW; 100% holding water capacity HWC, 25 °C), water deficit (WD; 40% HWC, 25 °C), high temperature (HT; 100% HWC, 40 °C) and water deficit plus high temperature (WD + HT; 40% HWC; 40 °C) conditions. (**A**) Partial least square-discriminant analysis (PLS-DA). (**B**) Variable importance in projection (VIP) scores of the PLS-DA model. Metabolites included in the VIP score lists have a score higher than 1, which indicates those that mostly contributed to the separation observed at the PLS-DA model of the respective cultivar. PLS-DA was carried out by combining data from all genotypes. The data were normalized by using Log and Auto-scaling transformations on the MetaboAnalyst platform^98^ (*n* = 5).

**Figure 8:**
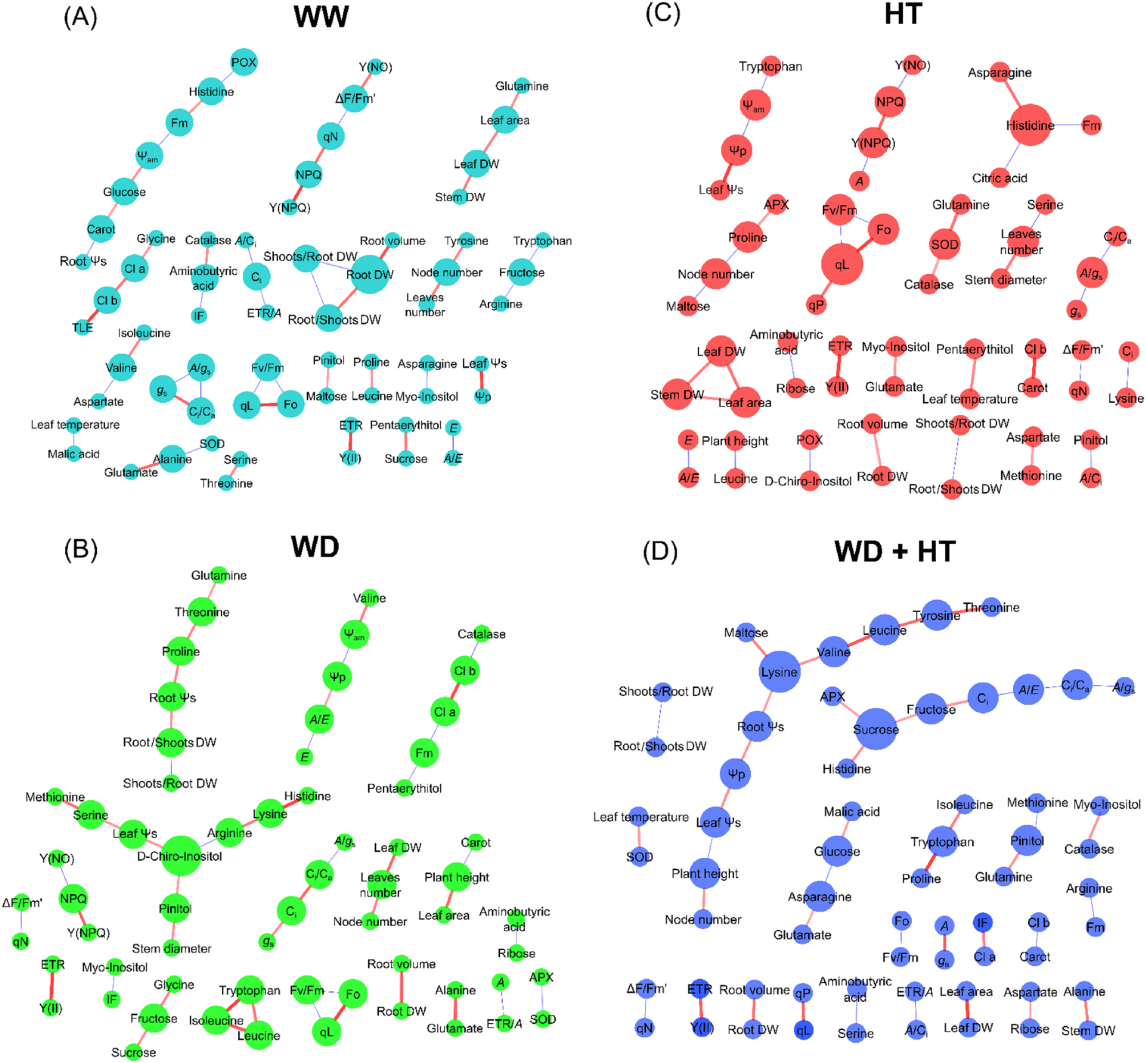
Correlation-based networks of soybean plants maintained for 8 days under (**A**) well-watered (WW; 100% holding water capacity HWC, 25 °C), (**B**) water deficit (WD; 40% HWC, 25 °C), (**C**) high temperature (HT; 100% HWC, 40 °C) and (**D**) water deficit plus high temperature (WD + HT; 40% HWC; 40 °C) conditions. The networks were created by combining data from anatomical, metabolic, and physiological levels. The nodes are the parameters and the link is the debiased sparse partial correlation coefficient (DSPC) among them, whenever is significant (*p* < 0.05). Red and blue links correspond to positive and negative correlations among the nodes. The thickness and the intensity of the colour of the links indicate a higher DSPC coefficient, in the module. Bigger nodes indicate a higher degree of connection. This analysis was performed using CorrelationCalculator software and the networks were designed by using MetScape on CYTOSCAPE software (*n* = 5).

We next integrated all data and compared the treatments by multivariate and correlation-based network analyses. PLS-DA analysis using all data revealed the same pattern previously observed using the metabolic data, in which WD + HT is separated from all other conditions by the first component, whilst HT was separated from WW and WD by the second component (Fig. 7). Network analysis revealed that the nodes are displaced into several subnetworks in all conditions. The subnetworks are mostly composed of nodes of the same scale of observation. For instance, nodes related to photochemical reactions (e.g. F_v_/F_m_, q_L,_ and F_0_) are connected to each other but not to any other parameter of the network. This is also observed for growth and gas exchange parameters, especially under WW conditions (Fig. 8A). However, certain metabolites are connected to important physiological parameters. For example, malate is negatively correlated to leaf temperature and Tyr and Gln are positively correlated with the number of nodes and leaf area, respectively, under WW conditions (Fig. 8A). By contrast, Gln is connected in a subnetwork that contains root Ψs and the DW ratios of root/shoots and shoots/roots under WD (Fig. 8B). Furthermore, whilst *A*/*E* was solely connected to *E* under WW and HT conditions (Fig. 8A,C), *A*/*E* was positively correlated to Ψp under WD conditions (Fig. 8B).

Under WD + HT conditions, *A*/*E* is part of a subnetwork composed of iWUE, *C*_i_, *C*_i_/*C*_a_, APX, and the metabolites sucrose, fructose, and histidine (Fig. 8D). Interestingly, *A* was not correlated to any parameter under WW condition, but it was negatively correlated to Y_NPQ_ under WD and HT conditions and strongly and positively correlated to *g*_s_ under WD + HT condition (Fig. 8A–D). Lys was found to be a hub connecting physiological (Ψ_w(am)_, root Ψ_s_, leaf Ψ_s_, plant height, and number of nodes) with maltose and amino acids (Val, Leu, Tyr, and Thr) under WD + HT conditions (Fig. 8D). The analysis of the network parameters revealed slight decreases and increases in network density and centralization under stress conditions, whilst the increase in network heterogeneity was more prominent under all stress treatments (Supplementary Table S1).

## Discussion

Identifying the mechanisms that contribute to the acclimation of soybean plants under abiotic stress conditions is pivotal for the selection of tolerant cultivars and to pave the way to obtaining new tolerant cultivars through genetic engineering of plant metabolism^36^. Here, soybean cultivars showed differential responses to WD, HT, and the combination of both stress conditions. Lower water potential has been described as a WD tolerance strategy, given that it entails the activation of osmotically compatible solute synthesis^37^, as observed here by the increases in sucrose, proline, and sugar alcohols in soybean plants under WD and WD + HT. Osmotic adjustment induces increased turgidity of plant cells, maintaining or at least minimizing the reduction in both plant growth and transpiration^38^, which, in turn, facilitate soil water uptake and leaf cooling, crucial for plants under WD and HT conditions. However, stomata, the master regulator of transpiration and photosynthesis, respond to a wide range of stimuli, not only plant water status^39,40^. Thus, despite negatively affecting photosynthetic performance^41^, reduced *g*_s_, which is closely associated to lower water potential, can represent a stress-avoidance mechanism^42^. Here, stomatal closure limited the transpiration rate of soybean plants, leading to an increase of 3.7 °C on average leaf temperature under WD + HT conditions. The heat stress per se did not affect the photosynthetic rate of soybean cultivars, suggesting that drought is a major stress factor for photosynthetic efficiency under WD + HT conditions. This idea is supported by the maintenance of *A*, *g*_s_, *E, A*/Ci, and Y_II_ values under HT conditions. Maintaining leaf temperature is further important to avoid protein denaturation and loss of membrane integrity^43^. However, plants need to balance the maintenance of photosynthesis with lower water loss through transpiration toward iWUE improvement, which was indeed observed here in soybean plants exposed to WD and WD + HT conditions.

Soybean plants under WD + HT had increased minimum fluorescence (F_0_) of dark-adapted leaves and reduced Y_II_ values, indicating damage to the antenna complex of the PSII^44^. To avoid excess energy-associated oxidative stress, plants generally activate protective mechanisms such as carotenoid accumulation^45^ and increased NPQ-mediated dissipation^46^. Nevertheless, these mechanisms were not effective in avoiding the reductions observed in Y_II_ and ETR, mainly in cultivars 7011 and 7209, in response to WD + HT. By contrast, no substantial differences were found in photochemical-related parameters in plants under HT conditions. High temperatures are related to an increased membrane fluidity, promoting changes in the assembling of membrane-inserted proteins^47^. Maintaining membrane stability is an important factor in stress tolerance. Indeed, the thermal stress tolerance of *Arabidopsis* plants has been attributed to their ability to maintain chloroplast membrane integrity^48^.

To avoid stress-induced oxidative imbalances, plants generally increase both the activity of antioxidant enzymes and the thermal dissipation of excess light absorbed via Y_NPQ_. The activation of Y_NPQ_ is competitive with photochemical processes, meaning that increased Y_NPQ_ may be followed by reductions in photochemical efficiency^49^. For instance, the increase in Y_NPQ_ in corn plants exposed to WD was accompanied by decreases in photosynthesis and plant growth^50^. Here, the increase in Y_NPQ_ was mainly observed in genotypes 7011 and 7209 under WD + HT. Interestingly, these genotypes have the lowest *A* and APX activity under WD + HT. This suggests that they are the most WD + HT-sensitive cultivars, as compared to Desafio and 7739. However, no substantial differences in plant growth and plant anatomy parameters were observed between the genotypes. These results indicate that the stress responses observed in 7011 and 7209 cultivars were sufficient to avoid deleterious effects on growth during the period analysed here. However, higher *A* and APX activity may confer a great advantage for Desafio and 7739 cultivars in longer periods of WD + HT, suggesting that total APX activity is a potential marker for breeding programs to obtain plants more tolerant to combined stresses. However, given that the APX family is composed of several isoforms that are located in different subcellular localizations and play different roles under stress conditions^51–53^, further studies aiming to unveil the contribution of soybean APX isoforms to WD + HT tolerance are now required to fully comprehend the role of APXs in plant acclimation/tolerance to WD + HT.

Abiotic stresses induce a wide range of metabolic changes in plants, which vary according to the species and the level of stress^12,54,55^. The availability of free amino acids generally increases stress tolerance in plants due to their participation in physiological mechanisms such as osmotic adjustment, ROS detoxification, intracellular pH regulation, and sustaining alternative mitochondrial respiratory pathways^56–59^. Here, PLS-DA and biomarker analyses revealed that several amino acids are implicated in the soybean responses to WD and HT, in which several of them have increased content in all stress conditions. Among these amino acids, arginine has structural importance for proteins, participates in nitrogen re-distribution, and is a precursor to the synthesis of polyamines and proline^60^. Proline was found in the VIP score list of PLS-DA, with a more prominent accumulation under WD + HT followed by WD condition. Proline accumulation contributes to maintaining the NADP^+^/NADPH ratio at levels adequate for cell metabolism^61^ and has been shown to be an important osmoregulatory mechanism for soybean acclimation to WD periods^62^. Indeed, proline was directly linked to root osmotic potential in the WD network, highlighting its role as an osmo-protectant.

Glycine is also known to be accumulated in response to WD in plants^29^. The level of glycine has been shown to increase in *Solanum lycopersicum* exposed to combined heat and salinity stress, acting as an osmo-protectant^63^. Similarly, increases in levels of the aromatic amino acid (tyrosine and tryptophan), as observed here in soybean plants, were also reported in wheat^64^, soybean^29^ and chickpea^65^ under drought conditions. These amino acids may act as alternative sources of metabolic energy^65^, and play important roles in ion transport, maintenance of water balance, and elimination of ROS^64^. The antioxidant effect of aromatic amino acids such as histidine prevents the intensification of damage from stress, as observed in drought-tolerant chickpeas^65^. The increase in isoleucine and leucine branched-chain amino acid levels in soybean plants may be a mechanism to provide carbon skeletons for respiration during stress, as was observed in several other species under different stress conditions, including WD^57,59,66^. In addition, increased asparagine content was observed here, which is important for maintaining osmotic pressure and translocating nitrogen^67^.

Our results also highlight that glutamine accumulation is important to plants under WD + HT. This amino acid was found in the PLS-DA VIP score list of all genotypes and identified as a WD + HT biomarker. Glutamine, in addition to its role in nitrogen metabolism, is an efficient signaling molecule related to metabolic regulation, stimulating defense responses in plants under stress conditions^68^. For instance, increased glutamine levels rapidly induced the expression of genes involved in the regulation of stress defense responses in rice plants^69^. Furthermore, glutamine metabolism is closely associated with the production of nitric oxide, proline, and GABA, which have been described as important components of plant stress responses^15,70^. Taken together, our results highlight the important role of amino acid metabolism in plant stress responses.

Beyond the changes in amino acids, the metabolism of sugars and polyols was altered under stress. Sugars and polyols are important for energy supply and act as osmoprotectors in plants^64,71^. Here, we observed that soybean cultivars under WD + HT resulted in increased levels mainly of sucrose and pinitol. Sucrose is the main product used in carbon distribution for non-photosynthetic tissues^72^, and, as polyols (pinitol and *myo*-inositol), act in the process of osmotic adjustment and the prevention of oxidative damage^58,73,74^. It’s important to note that we also observed an increase in *myo*-inositol in WD treatment but a decrease under WD + HT in soybean plants. The methylation of pinitol is linked to the *S*-adenosylmethionine (SAM) cycle^17^; thus, the synthesis of pinitol is linked to HT via photorespiration and would exhibit a demand for *myo*-inositol to be converted to pinitol in soybean plants. Furthermore, decrease maltose content was identified as a WD + HT biomarker and was connected to a subnetwork composed of amino acids and both root and leaf osmotic potentials. This suggests that the metabolism of maltose, which is closely associated with hexose and starch, is also important for soybean stress acclimation. This idea is supported by the fact that starch degradation is an important mechanism to provide substrates for the synthesis of osmo-protectants such as sugars, polyols, and proline under WD^75,76^. Therefore, decreased maltose content may be associated with a redirection of carbon flux from starch synthesis to starch degradation. In turn, starch-derived carbons could be used to sustain the synthesis of sugars, polyols, and amino acids in a source-limited conditions, i.e., in a period in which the photosynthetic rate is reduced, as observed in WD + HT conditions.

Stress responses involve changes at different levels, from epigenetic and gene expression to ultimately physiological and anatomical changes. Thus, in order to fully understand plant stress responses, is necessary to adopt non-reductionist approaches^77,78^. Systems biology analysis has the great advantage to overcome the barrier imposed by the reductionism perspective that simplifies complex mechanisms such as plant stress responses^79,80^. This is especially important considering that complex biological systems are defined by non-linear relationships and cannot be interpreted by the sum of their components^81,82^. Furthermore, emergent properties, which raise from the interaction among the components of the system, are not visualized by reductionism approaches^83^. Taking this into account, we have integrated metabolic, photosynthetic, and morpho-anatomical data through multivariate and correlation-based network analyses^84^. Interestingly, PLS-DA clearly separated the plants under WD + HT from plants under WW, WD, and HT. This strongly suggests that the combination of both stress factors is an emergent property of the system, in which the metabolic responses to the combination of WD + HT cannot be interpreted by solely summing the responses observed in these stresses separately.

The metabolic network analysis further corroborates this idea, given that the density, centralization, and heterogeneity of the metabolic network under WD + HT did not resemble the values observed under these stresses separately. Interestingly, substantial increases in network heterogeneity were observed in both metabolic and global networks under all stress conditions, when compared to WW plants. Additionally, plants under stress have a higher connection among components of different biological levels. For instance, whilst the subnetworks were mostly composed of nodes of the same level of organization (i.e., morphological, physiological, and metabolic) under WW conditions, the imposition of stress increased the connectivity of nodes from different levels. This is evidenced by the connections observed between different amino acids such as Ser, Asp, Leu, Glu, Gln, Arg, and Lys that belong to sub-networks composed of physiological and biochemical parameters under stress. Taken together, these results highlight that the stress conditions used here substantially alter the topology of both metabolic and the global network, in which the appearance of hub-like nodes, measured here through network heterogeneity, seems to be a response to stress. Higher network heterogeneity highlights the predominance of hubs in the network, i.e., the presence of nodes with a high degree of connection^85^. Hubs are important not only to change network topology but also to integrate different levels of the system, probably conferring a higher degree of stability to the system^83^.

## Conclusions

Water deficit negatively affected the metabolism of soybean plants, with an exacerbated effect when combined with high temperature. Metabolomics analysis highlight that the metabolic responses of plants under WD + HT differ substantially from both stresses isolated, which has several implications for molecular breeding toward WD + HT stress tolerance improvement in the current climate change scenario. Our results suggest that osmotic adjustment mainly from proline and pinitol accumulation associated with increased APX activity were important factors in the acclimation of soybean plants. We further demonstrate that increased amino acid content and decreased content of both maltose and *myo*-inositol were found as WD + HT biomarkers, highlighting the importance of amino acids and carbohydrate metabolisms for soybean stress acclimation.

## Methods

### Experimental conditions and experimental design

Soybean cultivars NS7209 IPRO (Nidera Seeds, São Paulo, Brazil; maturity group (MG) 7.2), NS7011 IPRO (Nidera Seeds, São Paulo, Brazil; MG 7.0), Desafio 8473 RSF (Brasmax Seeds, Passo Fundo, Brazil; 7.4), and 7739 M IPRO (Monsoy Seeds, São Paulo, Brazil; MG 7.7) were grown in growth chambers (Instalafrio, Pinhais, PR, Brazil) with controlled conditions of relative humidity (~ 65%), irradiance (~ 650 µmol m^−2^ s^−1^) and temperature (25/20 °C, day/night). The four soybean cultivars were selected due to their characteristics of high productivity and wide cultivation in the Centre-East of Brazil, which represents the major Brazilian region of soybean production. The genetic diversity of the genotypes used here is important to identify common metabolic and physiological responses to WD + HT.

Plants were grown in polyethylene pots containing 8 kg of substrate prepared from Red Latosol (LVdf) soil and sand (2:1). The substrate used had the following composition: pH CaCl_2_—5.6; P—0.7 mg dm^−3^; K—13.0 mg dm^−3^; Ca—1.54 cmol_c_ dm^−3^; Mg—0.22 cmol_c_ dm^−3^; Al—0.05 cmol_c_ dm^−3^; H^+^ Al—1.3 cmol_c_ dm^−3^; S—3.5 mg dm^−3^; B—0.8 mg dm^−3^; Cu—1.0 mg dm^−3^; Fe—37.8 mg dm^−3^; Mn—13.2 mg dm^−3^; Zn – 0.1 mg dm^−3^; Na—6.0 mg dm^−3^; SB—58%; CTC—3.1 cmol_c_ dm^−3^; organic matter—109%. Based on these characteristics, liming was performed using dolomitic limestone, increasing the base saturation to 60%. The plants were fertilized with 0.2 g dm^−3^ of mono-ammonium phosphate (MAP), 0.16 g dm^−3^ of potassium chloride (KCl), 0.18 g dm^−3^ of potassium sulphate (K_2_SO_4_), 0.2 g dm^−3^ of urea (CH_4_N_2_O) and 0.026 g dm^−3^ of zinc sulphate (ZnSO_4_), according to the recommendation for Cerrado soils in which soybean are cultivated^86^.

Treatments consisted of a combination of two water replacements (100 and 40% of soil holding water capacity, HWC) and two temperatures (25/20 °C or 40/25 °C, day/night) imposed at the V3 development stage. The control of soil moisture in the pots was done using the gravimetric method, by replacing the water lost by evapotranspiration on a daily basis. The high temperature (HT) was imposed when the plants reached the water deficit by gradually increasing from 25 °C at 10 h until reaching 40 °C ± 0.5 °C at 12:00 h, maintained for 5 h. After this period, the temperature gradually decreased until it returned to 25 °C around 19:30 h, repeating this cycle in the following day. Thermal treatments were imposed for an 8-day period, after which physiological, biochemical, metabolic, and morphoanatomical evaluations were made.

The experimental design was arranged in randomized blocks, in a factorial design with two water replacements (100% and 40% HWC) and two temperatures (25 °C and 40 °C), with five replicates. Two plants were grown per pot, representing one experimental unit. One plant was used for gas exchange and growth measurements and the other for biochemical and molecular analyses.

### Water relations and leaf temperature

Leaf water potential (Ѱ_w(am)_) was measured using a Scholander pressure chamber (Model 3005-1412, Soilmoisture Equipment Corp., Goleta, CA, USA). Osmotic leaf (Ѱ_s(leaf)_) and root (Ψ_s(root)_) potentials were determined using a vapor pressure osmometer (VAPRO 5600, ELITech, Puteaux, France) and calculated using the Van’t Hoff equation, expressed as MPa. Leaf temperature was measured using a digital infrared thermometer (Model TI-920, Instrutherm Ltda, São Paulo, SP, Brazil), approximately 15 cm from the leaf limb. The measurements were performed at 09:00 h, with the chamber at 25 °C.

### Gas exchange and chlorophyll *a* fluorescence analysis

Gas exchange was measured in fully expanded leaves to determine the photosynthetic rate (*A*, µmol CO_2_ m^−2^ s^−1^), stomatal conductance (*g*_s_, mol H_2_O m^−2^ s^−1^), transpiration rate (*E*, mmol H_2_O m^−2^ s^−1^), and the ratio of internal and external CO_2_ concentration (*C*_*i*_*/C*_*a*_). We further estimate intrinsic water-use efficiency (*A*/*g*_s_) and instantaneous carboxylation efficiency (*A*/*C*_i_,). The measurements were performed using an infrared gas analyzer (IRGA, LI-6400xrt, Licor®, Nebraska, USA), under constant photosynthetically active radiation (*PAR*, 1000 µmol m^−2^ s^−1^) at the environmental CO_2_ concentration (~ 430 µmol mol^−1^), temperature (~ 25 °C), and relative humidity (~ 65%). Chlorophyll *a* fluorescence was measured using the IRGA coupled to a leaf chamber fluorometer (6400xt, Licor®, Nebraska, USA). The minimal chlorophyll fluorescence (F_0_) and maximum quantum yield of photosystem II (PSII) (Fv/Fm) were measured after 30 min of dark adaptation. In light-adapted leaves, the apparent electron transport rate (ETR), the fraction of opened PSII reaction centers (qL), the effective quantum yield of PSII (Y_II_), and the yield of non-photochemical quenching (Y_NPQ_) were obtained.

### Determination of antioxidant enzyme activities

To determine the activities of superoxide dismutase (SOD), catalase (CAT), ascorbate peroxidase (APX), and total peroxidase (POX), fresh leaf tissue was homogenized in potassium phosphate buffer solution (pH 6.8). SOD activity was determined by measuring the ability to photochemically reduce *p*-nitrotetrazolium blue (NBT), at 560 nm in a spectrophotometer (Evolution 60S, Thermo Fisher Scientific Inc., MA, USA), according to (Del Longo et al.^87^), considering that one SOD unit was defined as the amount of enzyme required to inhibit NBT photoreduction by 50%. CAT activity was assayed according to the method described by Havir and McHale^88^ and calculated as the rate of hydrogen peroxide (H_2_O_2_) decomposition at 240 nm for 3 min at 25 °C, using a molar extinction coefficient of 36 M^−1^ cm^−1^. APX activity was determined according to Nakano and Asada^89^ and was measured as the rate of ascorbate oxidation at 290 nm within 1 min at 25 °C, using a molar extinction coefficient of 0.0028 M^−1^ cm^−1^. POX activity was measured following the method described by Kar and Mishra^90^. Purpurogallin production was determined by increasing the absorbance of the reaction at 420 nm for 1 min at 25 °C, using the extinction coefficient of 2.47 mM^−1^ cm^−1^^91^. Enzyme activity was expressed based on protein, the concentration of which was determined according to the Bradford method.

### Leaf morphoanatomical characterization

Samples (~ 3 cm^2^) from the middle region of the last fully expanded leaf were collected and fixed in Karnovsky solution. After 24 h, the material was pre-washed in phosphate buffer and dehydrated in a gradual ethyl alcohol series, pre-infiltered and infiltered in historesin (Leica, Germany), according to the manufacturer’s recommendation. Samples were then transversely sectioned to 5-μm thickness in a rotating microtome (1508R model, Logen Scientific, China). Sections were stained with toluidine blue (0.05% in 0.1 M phosphate buffer, pH 6.8), and anatomical observations of the adaxial and abaxial epidermis, palisade, and spongy parenchyma and mesophyll were made using images photographed with an Olympus microscope (BX61, Olympus, Tokyo, Japan) coupled to a DP-72 camera using the light-field option. The micromorphometry measurements were obtained from the previous images using ImageJ software (Image Processing and Analysis in Java, v.147, USA). Ten observations per replicate were measured for each structure evaluated.

### Growth analysis

Growth parameters such as plant height (PH, cm), stem diameter (SD, mm), and leaf area (LA) were determined. Shoot (leaves and stem) and roots were separated and dried at 65 °C for 72 h to obtain the shoot dry matter (SDM, g) and root dry matter (RDM, g). The ration RDM/SDM was also calculated.

### Metabolic extraction and analysis

Leaf samples were collected and immediately immersed in liquid nitrogen, and later homogenized and lyophilized. For metabolites extraction, the samples were microwave dried to prevent metabolic turnover^92^ and then approximately 40 mg of dry leaf samples were extracted in methanol/chloroform/water (12:5:3, v/v/v) at 75 °C for 30 min. The water fraction of the extraction mixture consisted of a 0.1% solution of internal standard. Samples were centrifuged and the supernatants were collected and mixed with chloroform and Milli-Q water to facilitate phase separation. The water–methanol soluble fractions were collected and stored at − 20 °C for further analysis.

Analysis of soluble carbohydrates, sugars, and organic acids were performed using gas chromatography (Agilent 7890A) coupled to a triple quadrupole mass spectrometer (7000 Agilent Technologies Inc, Santa Clara, CA, USA). An HP5 column (0.25-mm internal diameter, 30-m long, 0.25-µm film thickness) was used for chromatographic separation. Extracts were dried down in a SpeedVac and resuspended in 400ul of anhydrous pyridine. Samples were then derivatised using a 1:10 mixture of N,O-Bis(trimethylsilyl)trifluoroacetamide (BSTFA): trimethylcholorsilane (TMCS). Samples were incubated for 35 min at 75 °C and were analysed using GC–MS within 24 h. A 20:1 split injection was made at 300 °C initial oven temperature program of 60 °C for 2 min, ramping to 220 °C at 10 °C min^−1^ (hold for 5 min) then ramping at 10 °C min^−1^ to 300 °C (hold for 5 min). Peak integration was made using Agilent MassHunter software (Agilent). Metabolites were identified based upon retention time and mass fragment comparisons within the MassHunter METLIN Metabolomics database (Agilent Technologies, Santa Clara, CA USA). A mixed standard was made from a stock solution containing 500 µg mL^−1^ of each analyte. Appropriate aliquots were taken to make standard concentrations between 0.5 and 50 µg mL^−1^. The results were expressed based on the dry weight.

Analysis of amino acids was performed using the underivatized extracts on a 1290 Infinity liquid chromatography (LC)-MS system (6520 QTOF, Agilent Technologies Inc, Santa Clara, CA, USA). A 3.5 µL sample was injected into a Zorbax SB-C18 column (2.1 × 150 mm, 3.5 µm) and separation was achieved by gradient elution with water and methanol. The QTOF was tuned to operate at the low-mass range (< 1700 AMU). Data acquisition was performed in scan mode (60–1700 m/z) and ionization performed in positive ion mode. Peaks were integrated and their relative quantities were calculated using MassHunter software (Agilent®). A mixed standard was made from a stock solution containing 500 µg mL^−1^ of each analyte. The solutions were kept frozen at − 20 °C. Appropriate aliquots are taken to make resulting standard concentrations between 0.1 and 20 µg mL^−1^. Metabolites were identified based on their retention times relative to standards as well as their formula mass. The results were expressed based on the dry weight.

### Metabolic network analysis

Metabolomics and physiological parameters data were used to create correlation-based networks, in which the nodes are the metabolites and the links are the strength of debiased sparse partial correlation (DSPC) coefficient (*r*)^93^. The correlation-based networks were created by using Metscape on CYTOSCAPE^94,95^ and limiting the significance of the correlation between nodes to *p* < 0.05 or by restricting *r* values to − 0.5 < *r* > 0.5^96^. The parameters of network density, network heterogeneity, and network centralization were obtained using the java plugin NetworkAnalyzer on CYTOSCAPE software^97^.

### Statistical analysis

The data were subjected to factorial analysis of variance and the means were compared using the Tukey test (*p* < 0.05), using Analysis System Program Variance (SISVAR, version 5.4). The metabolomics data were analysed by multivariate analysis such as partial least square-discriminant analysis (PLS-DA) and biomarker analysis based on receiver operating characteristic (ROC) curves using the MetaboAnalyst platform^98^. Only metabolites with VIP scores higher than 1 were considered to have an important impact on the PLS model, according to recommendations of metabolomics data^99^. The VIP score figures only demonstrate metabolites with a VIP score > 1. These metabolic analyses were normalized by using Log and Auto-scaling transformations on MetaboAnalyst.

### Research involving plants statement

This study was developed with commercial seeds, therefore non-exotic or at risk of extinction, under controlled conditions, meeting all institutional, national and international guidelines and legislation for cultivated plants.

## Supplementary Information

Supplementary Figures.

## Data Availability

All data generated or analysed during this study are included in this published article (and its Supplementary Information files).
