# Supplementary material for: Metabolic, physiological and anatomical responses of soybean plants under water deficit and high temperature condition
Source: Sci Rep. 2022 Oct 1;12:16467. doi: 10.1038/s41598-022-21035-4 (PMC9526742; doi:10.1038/s41598-022-21035-4)
Supplement: Supplementary file 1 — Supplementary Figures. [file 41598_2022_21035_MOESM1_ESM.docx]

### **Supplementary Material**

**Table S1.** Network parameters from metabolic and correlation-based networks integrating anatomical, metabolic and physiological data. Metabolic network (metabolite *vs* metabolite) corresponds to a network in which the metabolites are the nodes and the links are the correlation among them. The other network (physiological parameter *vs* metabolite) corresponds to a network created by correlating metabolites with physiological and biochemical parameters. All network parameters were obtained by Network Analysis in Cytoscape (Assenov *et al.*, 2008).

| **Network parameter** | **Metabolite vs metabolite** | | | |  | **Physiological parameter vc metabolite** | | | |
| --- | --- | --- | --- | --- | --- | --- | --- | --- | --- |
|  | **WW** | **WD** | **HT** | **WD+HT** |  | **WW** | **WD** | **HT** | **WD+HT** |
| Network density | 0.032 | 0.044** | 0.030* | 0.037** |  | 0.017 | 0.016* | 0.014* | 0.014* |
| Network centralization | 0.076 | 0.064* | 0.042* | 0.071* |  | 0.024 | 0.025** | 0.027** | 0.026** |
| Network heterogeneity | 0.674 | 0.704** | 0.828** | 0.723** |  | 0.535 | 0.604** | 0.688** | 0.634** |

*decreased in relation to WW

### **increased in relation to WW

**Figure S1.** Leaf anatomical characterization of soybean plants maintained for eight days under well-watered (WW; 100% holding water capacity HWC, 25ºC), water deficit (WD; 40% HWC, 25ºC), high temperature (HT; 100% HWC, 40ºC) and water deficit plus high temperature (WD+HT; 40% HWC; 40°C) conditions. A) Palisade parenchyma thickness (µm). B) Spongy parenchyma thickness (µm). C) Mesophyll cell thickness (µm). D) leaf thickness (µm). E) Epidermis adaxial thickness (µm). F) Epidermis abaxial thickness (µm). Bars represent mean ± SE (*n* = 5). Means followed by the same uppercase letters compare cultivars within the same water and temperature treatment. Means followed by the same lowercase letters compare water and temperature treatments within the same cultivar, as determined by Tukey test at 5% of probability.

### **Figure S2.** Growth parameters of soybean plants maintained for eight days under well-watered (WW; 100% holding water capacity HWC, 25ºC), water deficit (WD; 40% HWC, 25ºC), high temperature (HT; 100% HWC, 40ºC) and water deficit plus high temperature (WD+HT; 40% HWC; 40°C) conditions. A) Plant height (PH, µm). B) Stem diameter (SD, mm). C) Leaf area (LA, cm^2^). D) Shoot dry matter (SDM, g). E) Root dry matter (RDM, g). F) Ratio between RDM and SDM (RDM/SDM). Bars represent mean ± SE (*n* = 5). Means followed by the same uppercase letters compare cultivars within the same water and temperature treatment. Means followed by the same lowercase letters compare water and temperature treatments within the same cultivar, as determined by Tukey test at 5% of probability.


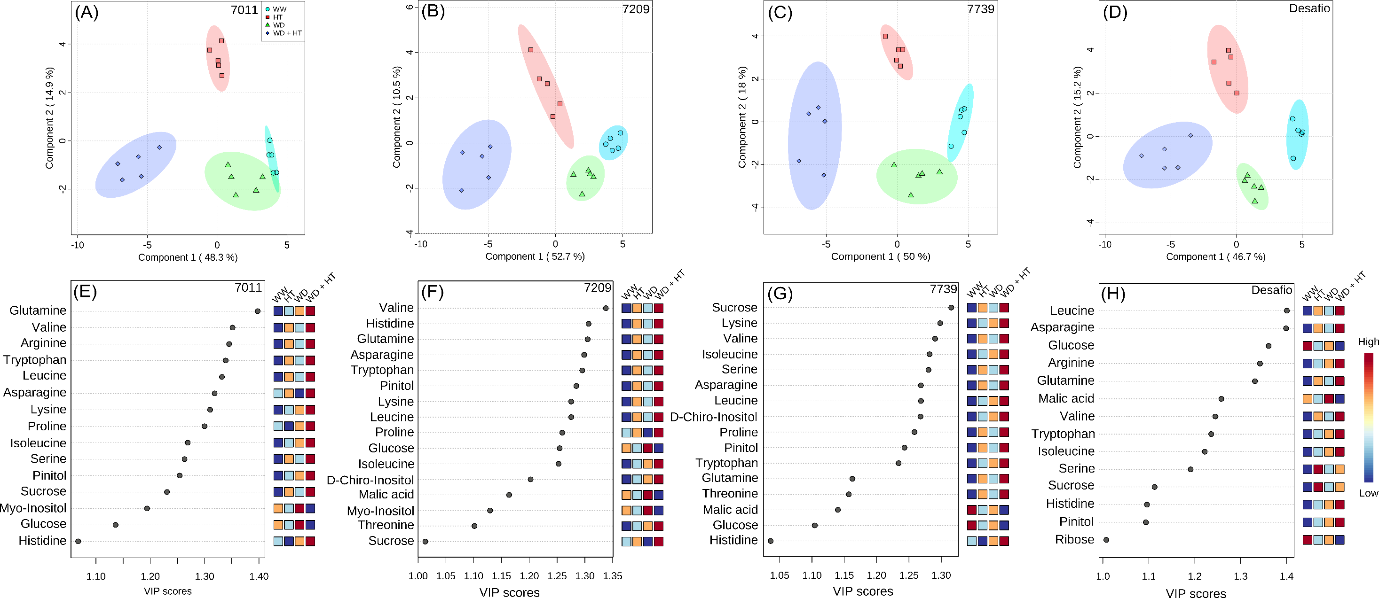


**Figure S3.** Cultivar-specific metabolic characterization of soybean plants maintained for eight days under well-watered (WW; 100% holding water capacity HWC, 25ºC), water deficit (WD; 40% HWC, 25ºC), high temperature (HT; 100% HWC, 40ºC) and water deficit plus high temperature (WD+HT; 40% HWC; 40°C) conditions. A-D) Partial least square-discriminant analysis (PLS-DA). E-H) Variable importance in projection (VIP) scores of the PLS-DA model. Metabolites included in the VIP score lists have VIP score higher than 1, which indicates those that mostly contributed to the separation observed at the PLS-DA model of the respective cultivar. PLS-DA was carried out combining data from all genotypes. The data was normalized by using Log and Auto-scaling transformations on the MetaboAnalyst platform (Chong *et al.*, 2018) (*n* = 5).


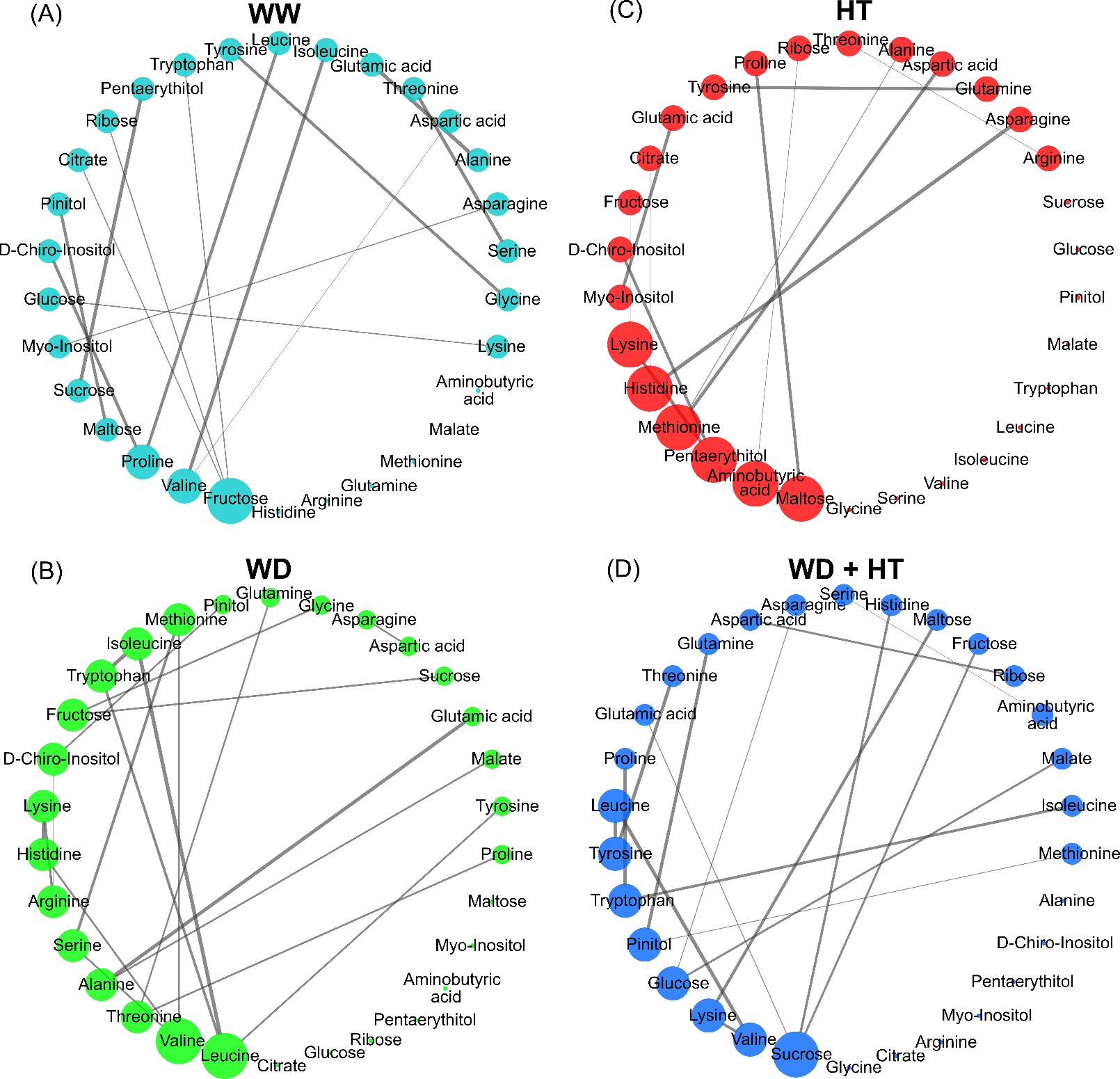


**Figure S4.** Metabolic networks of soybean plants maintained for eight days under A) well-watered (WW; 100% holding water capacity HWC, 25ºC), B) water deficit (WD; 40% HWC, 25ºC), C) high temperature (HT; 100% HWC, 40ºC) and D) water deficit plus high temperature (WD+HT; 40% HWC; 40°C) conditions. The networks were created using metabolite profiling data from all genotypes. The nodes are the parameters and the link is the debiased sparse partial correlation coefficient (DSPC) among then, whenever is significant (*P* < 0.05). Thicker arrows indicate higher DSPC coefficient, in module. Bigger nodes indicate higher degree of connection. This analysis was performed using CorrelationCalculator software and the networks designed by using MetScape on CYTOSCAPE software (*n* = 5).

**
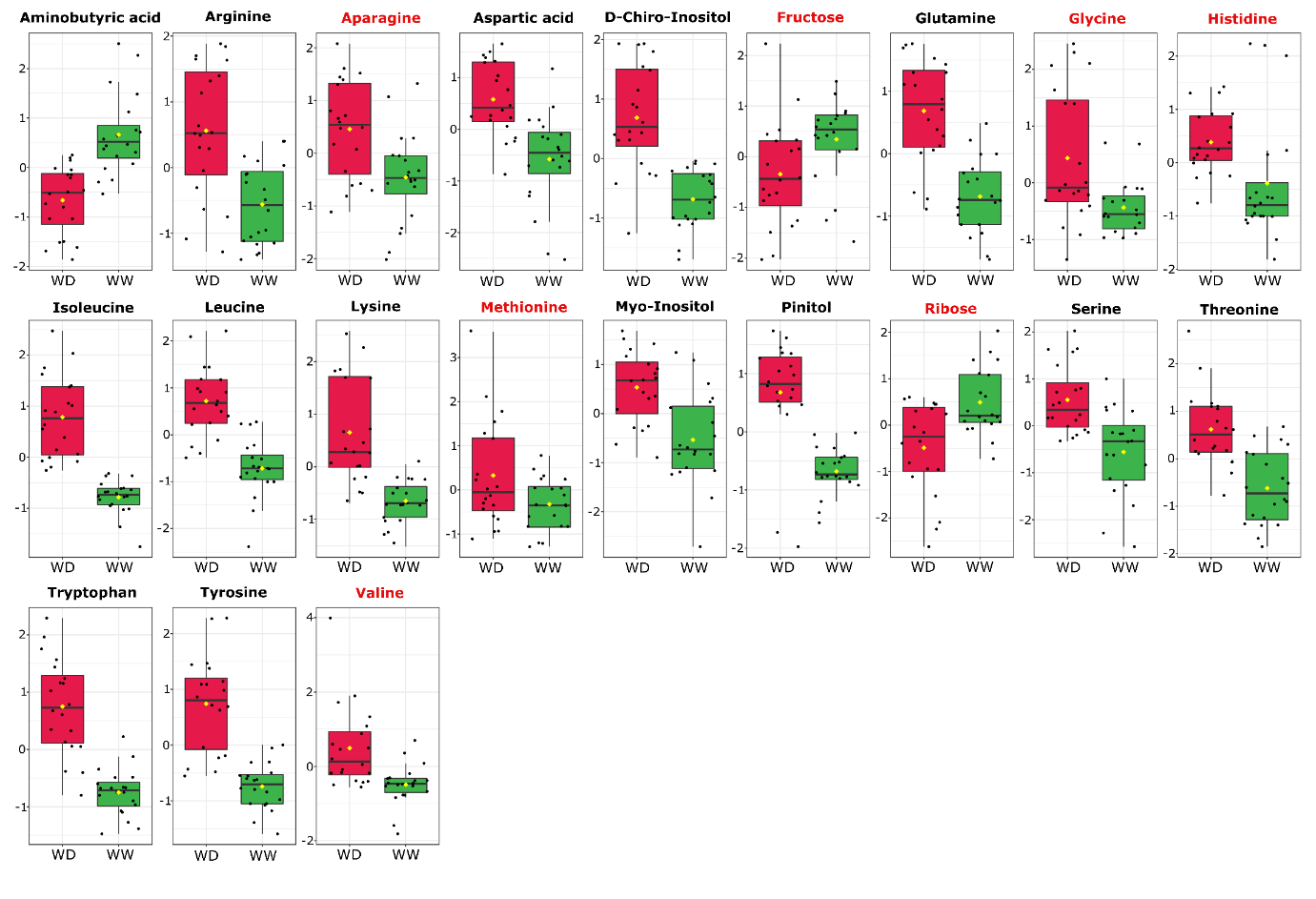
**

**Figure S5.** Box plots of metabolites identified as water deficit (WD) biomarkers. Green and red box plots indicate the relative content of metabolites found in soybean plants under well-watered (WW) and WD conditions, respectively. Biomarkers were identified based in receiver operating characteristic (ROC) curves using Log and Auto-scaling normalized data on the MetaboAnalyst platform (Chong *et al.*, 2018). Metabolites in black and red indicate statiscal difference at *P* < 0.001 and *P* < 0.05, respectively.

**
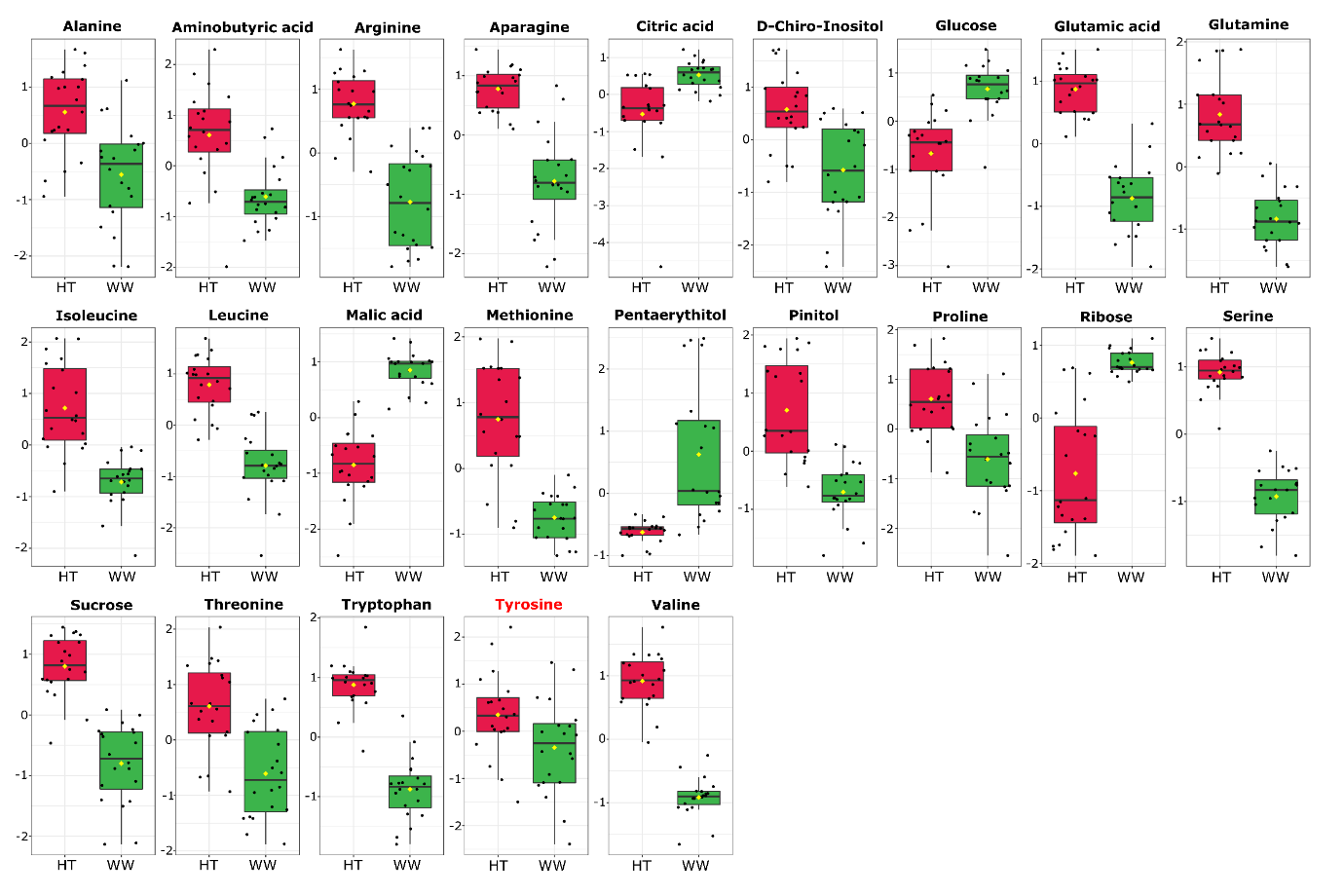
**

### **Figure S6.** Box plots of metabolites identified as biomarkers in the high temperature (HT) treatment. Green and red box plots indicate the relative content of metabolites found in soybean plants under well-watered (WW) and HT conditions, respectively. Biomarkers were identified based in receiver operating characteristic (ROC) curves using Log and Auto-scaling normalized data on the MetaboAnalyst platform (Chong *et al.*, 2018). Metabolites in black and red indicate statiscal difference at *P* < 0.001 and *P* < 0.05, respectively.

**
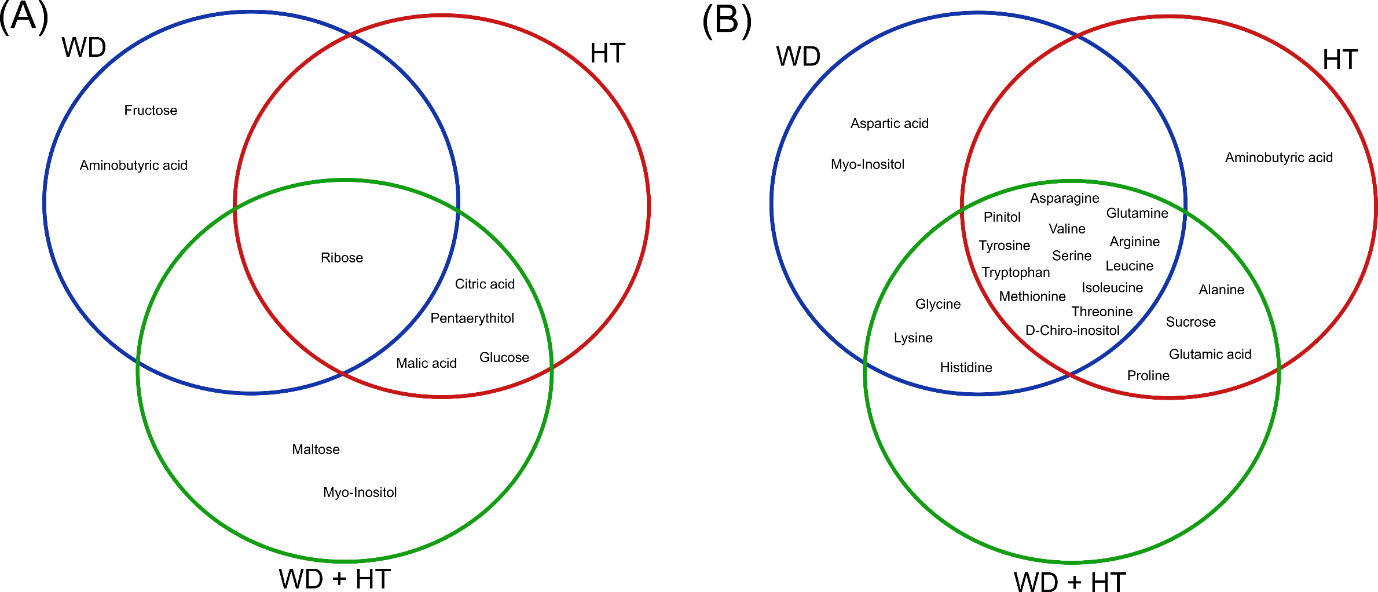
**

**Figure S7.** Veen diagram highlighting the overlap among the biomarkers found in soybean plants under water deficit (WD), high temperature (HT) and water deficit plus high temperature (WD+HT) conditions. These metabolites correspond to those shown at the Figures S2-4. The metabolites are separated in two groups, those that have A) decreased or B) increased content in each stress condition, when compared to well-watered plants.


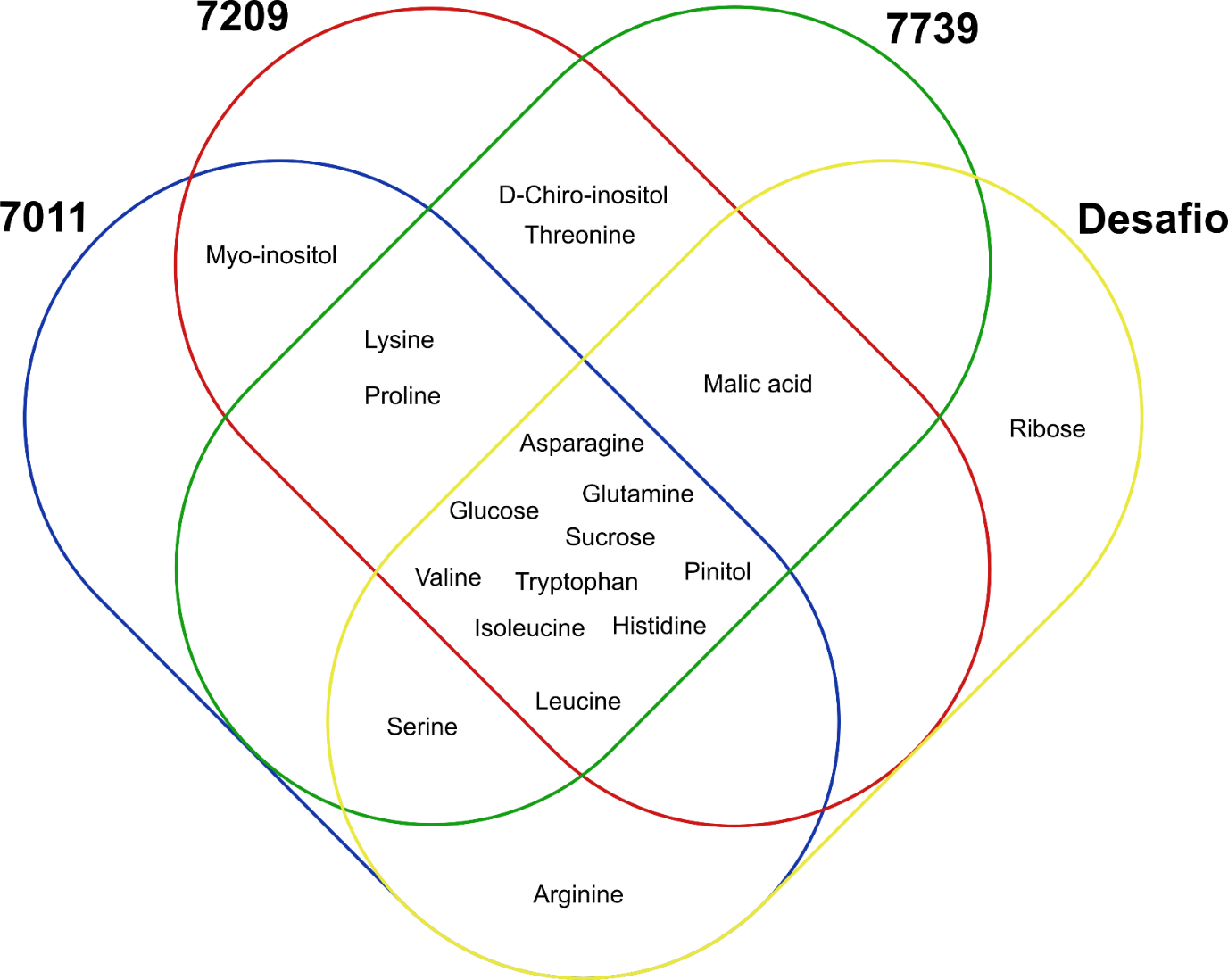


**Figure S8.** Veen diagram highlighting the overlap among the metabolites found in the VIP score list of the PLS-DA of each cultivar. These metabolites correspond to those shown at the Figure 8.
